# Nutritional Quality and Oxidative Stability during Thermal Processing of Cold-Pressed Oil Blends with 5:1 Ratio of ω6/ω3 Fatty Acids

**DOI:** 10.3390/foods11081081

**Published:** 2022-04-08

**Authors:** Dominik Kmiecik, Monika Fedko, Aleksander Siger, Przemysław Łukasz Kowalczewski

**Affiliations:** 1 Department of Food Technology of Plant Origin, Poznań University of Life Sciences, Wojska Polskiego 31, 60-634 Poznan, Poland; przemyslaw.kowalczewski@up.poznan.pl; 2 Department of Gastronomy Science and Functional Food, Poznań University of Life Sciences, Wojska Polskiego 31, 60-634 Poznan, Poland; monika.fedko@up.poznan.pl; 3 Department of Food Biochemistry and Analysis, Poznań University of Life Sciences, Wojska Polskiego 31, 60-634 Poznan, Poland; aleksander.siger@up.poznan.pl

**Keywords:** bioactive compounds, cold-pressed oils, fatty acids, oil quality, oxidative stability, tocopherols, heating of oils, nutritional quality

## Abstract

The growing awareness of consumers means that new products are sought after, which, apart from meeting the basic demand for macronutrients and energy, will have a positive impact on our health. This article is a report on the characteristics of the new oil blends with a nutritious ω6/ω3 fatty acid ratio (5:1), as well as the heat treatment effect on the nutritional value and stability of the oils. Prepared oil blends were heated at 170 and 200 °C. The fatty acid composition and the changes in tocochromanols content during heating were analyzed, as well as the formation process of polar compounds and triacylglycerol polymers. During heating the highest loss of tocochromanols was characteristic of α-tocopherol and α-tocotrienol. The total content of tocopherols after heating was reduced to 1–6% of the original content in the unheated oil blends. The exception was the blend of oil with wheat germ oil, in which a high content of all tocopherols was observed in unheated and heated samples. The content of the polar fraction during heating increased on average 1.9 and 3.1 times in the samples heated at 170 and 200 °C, respectively, compared to the unheated oils. The level of the polar fraction was related to the high content of tocopherols or the presence of tocopherols and tocotrienols in the heated sample. The polymerization of triacylglycerols led mainly to the formation of triacylglycerol dimers. Trimers were observed in a small number of heated samples, especially those heated at 200 °C. Regardless of the changes in heated oils, none of the prepared blends exceeded the limit of the polar fraction content, maintaining the programmed ratio of ω6 to ω3 acids. The principal component analysis (PCA) used to define the clusters showed a large variety of unheated and heated samples. An outlier in all clusters was a blend of oil with wheat germ oil. In these samples, the degradation of tocopherols molecules and the increase of triacylglycerol polymers and the polar fraction content were the slowest.

## 1. Introduction

Oils and fats are important components of the diet, providing not only a significant portion of energy but also lipophilic vitamins (A, D, E, and K) and bioactive compounds [1,2]. The vast majority of the fats we consume are fats of plant origin [3], which are characterized by a high content of monounsaturated (MUFA) and polyunsaturated fatty acids (PUFA) [4]. The growing interest of consumers in low-processed foods with high nutritional value makes cold-pressed oils in particular demand. In this process, oil is pressed from the pre-cleaned seeds under controlled temperature conditions, keeping the screw press temperature below 40 °C. Low temperature reduces the loss of MUFA and PUFA and also reduce the activity of seed enzymes and the extraction of unwanted compounds from the seeds into the oil [5,6]. Although cold-pressed oils have a high level of PUFA, it should be noted that PUFA oxidation is the main reaction that degrades the quality of the oil. Depending on the origin and type of oil, they are characterized by different oxidation stability, especially during thermal processing of food [7,8,9]. PUFAs have a well-documented protective effect against cardiovascular diseases and cancer, but products resulting from their oxidation raise concerns about possible negative health effects [10].

There are currently no clear recommendations regarding the consumption of *n*-6 and *n*-3 fatty acids. Depending on the country and even the diet used, the recommendations for different groups of the population vary. The WHO/FAO has suggested an optimal *n*-6/*n*-3 ratio of 5:1–10:1 [11], while the recommendations of organizations in different countries sometimes issue different recommendations, e.g., Sweden recommends ratio 5:1, Canada 4:1–10:1, and Japan 2:1 [12]. Analyzes of food products show a significant imbalance in the *n*-6/*n*-3 ratio, sometimes reaching a ratio above 20:1 [13,14,15]. Excessive consumption of *n*-6 fatty acids can cause negative health effects, as it promotes inflammation, which can lead to heart disease and other diseases [16]. Omega-3 fatty acids modulate prostaglandin metabolism and reduce the level of triglycerides, when consumed in high doses, they effectively lower cholesterol and also have anticoagulant and anti-inflammatory properties [15]. Plant-based vegan diets are gaining popularity, especially among young people. It seems particularly important to balance the *n*-6/*n*-3 ratio in more and more widely used plant diets. According to the literature, vegans and vegetarians consume less -linolenic acid (ALA) than linoleic acid (LA), which limits the conversion of ALA to eicosapentaenoic acid (EPA) and docosahexaenoic acid (DHA) [17]. Furthermore, a vegetarian diet is deficient in EPA and DHA [18,19].

Biological activity, including antioxidant capacity, is an important function of food. Antioxidant compounds supplied with food protect cells against the harmful effects of reactive oxygen species. They can effectively prevent many civilization diseases, including cardiovascular diseases, obesity, obesity-related metabolic disorders, cancers, gastrointestinal disturbances, or neurodegenerative dysfunctions [20]. In addition, antioxidants have been shown to prevent plaque build-up in the arteries, preventing future cardiovascular problems [21,22]. Biologically active compounds present in oils, such as carotenoids, polyphenols, phytosterols, and tocochromanols, have a strong antioxidant effect and thus can protect PUFA against oxidation [2,23,24]. Tocochromanols (tocopherols and tocotrienols) are fat-soluble molecules belonging to the group of vitamin E compounds [25]. They play an important role in human nutrition but are also an important inhibitor of oxidative changes in oils [26]. The main role of tocopherols and tocotrienols as antioxidants is believed to be the scavenging of lipid peroxide radicals, which are responsible for the propagation of lipid peroxidation [27]. Another group of antioxidant compounds commonly found in cold-pressed oils are polyphenols [7]. They include derivatives of benzoic and cinnamic acid and can be divided into two main groups: non-flavonoids and flavonoids [28]. Polyphenols act against lipid oxidation due to hydrogen donation and sequential radical quenching [29].

In the qualitative assessment of edible oils, an important role is played by their oxidative stability, which determines their technological suitability [30]. Oxidation is a complex and multi-step process that is induced by many different factors, including oxygen, heat, free radicals, light, and even the presence of selected metal ions [31]. From the point of view of food production, its preservation and preparation for consumption, stability during heating seems to be extremely important. Among the many food preparation methods, frying is one of the most popular due to the unique structure, taste, and aroma of the fried product. Furthermore, the frying process is quick and inexpensive [32]. High temperatures during frying, typically in the 170–180 °C range, long periods of use of the oil, and even the type of product to be fried all have a significant impact on the degradation of the oil used for frying [33,34]. As a consequence, various reactions that reduce the quality of the oil are observed, as well as lead to the formation of potentially harmful compounds. The typical chemical reactions observed during deep-fat frying can be divided into hydrolysis, oxidation, isomerization, and polymerization [35]. Many methods have been described and proposed for assessing the quality of frying fats during frying. Total polar compounds and polymer triglycerides are proposed as the most reliable methods of monitoring fat changes [36].

To date, there is little literature data on oil blends with programmed ω6/ω3 acid ratio, especially using cold-pressed oils to obtain them. There is also a lack of data on changes in the time of heat treatment commonly used in food production. Considering the above, the aim of this study was to design and characterize oil blends with 5:1 ratio of ω6/ω3 fatty acids and to evaluate the stability and changes during heating process. The content of tocochromanols, polar compounds, and polymers of triacylglycerols was also assessed.

## 2. Materials and Methods

### 2.1. Materials

The basic materials for the research were seven pressed and one refined oil commercially purchased. The cold-pressed oils were: rapeseed oil, evening primrose oil, camelina oil, black cumin oil, hemp oil, linseed oil, and wheat germ oil. The rice bran oil was a refined oil. All oils were packed in dark glass bottles and stored in a refrigerator at +3 °C until oil blends were prepared. From basic oils, 8 blends were prepared. The prepared blends were characterized by 5:1 ratio of ω6/ω3 fatty acids. The blends were prepared by mixing the appropriate oils in a specific proportion in accordance with Polish patent applications [37,38]. The composition of the prepared oil blends is shown in Table 1.

### 2.2. Heating Procedure

Each blend of oils was heated in a thin layer using a steel pan with a diameter of 20 cm. 50 mL of oil was transferred to the pan and heated at 170 °C and 200 °C ± 5 °C. Two different temperatures were chosen to determine the dynamics of changes in oil degradation. The temperature of 170 °C is often chosen as a compromise between the fast frying process, appropriate sensory features, low-fat content, and fat degradation. Heating in a thin layer promotes uncontrolled overheating of the oil, and a slight increase in temperature can lead to a sharp change in the rate of oil degradation. Heating process was carried out in two independent replications. The magnetic stirrers (IKA RET basic, MS-H-Pro, IKA Works, Inc., Wilmington, NC, USA) were used for the heating process. The temperature was controlled throughout the heating using an electronic thermometer. The heating cycle consisted of two stages: heating to the set temperature and then maintaining the temperature for 10 min. When the heating temperature was 170 °C the first step lasted 7 min, and when the heating temperature was set at 200 °C the first step lasted 9 min. After heating, the oil samples were sealed under nitrogen in dark glass bottles and kept frozen at −24 °C until analysis.

### 2.3. Fatty Acid Composition Analysis

The fatty acid composition was determined according to the AOCS Official Method Ce 1 h-05 [39] using an Agilent 7820A GC equipped with a flame ionization detector (FID) (Agilent Technologies, Santa Clara, CA, USA). The oil samples were first dissolved in hexane and transesterified with sodium methylate. After *trans*-esterification, the fatty acid methyl esters (FAME) were separated using the SLB-IL111 capillary columns (Supelco, Bellefonte, PA, USA) (100 m, 0.25 mm, 0.20 mm). The conditions during the analysis were as follows: the initial oven temperature was 150 °C and it was increased to 200 °C at 1.5 °C/min; the injector and detector temperatures were 250 °C; split 1:10; the carrier gas was helium at 1 mL/min. The FAME were identified by comparison with retention times of commercially available standards grain fatty acid methyl ester mix (Supelco, Bellefonte, PA, USA). The results were expressed as a percentage of total fatty acids.

### 2.4. Tocochromanols Analysis

The tocochromanols (tocopherols, tocotrienols, and plastochromanol-8 (PC-8)) content was determined according to Siger et al. [40]. The tocochromanols content was analyzed using a Waters HPLC system (Waters, Milford, MA, USA) equipped with a LiChrosorb Si 60 column (250 × 4.6 mm, 5 μm, Merck, Darmstadt, Germany), a fluorimetric detector (Waters 474), and a photodiode array detector (Waters 2998 PDA). The conditions during the analysis were as follows: mobile phase was a mixture of n-hexane with 1.4-dioxane (96:4, *v:v*); the flow rate was 1.0 mL/min; injection sample volume was 10 mL; the excitation wavelength was set at ʎ = 295 nm and the emission wavelength at ʎ = 330 nm. The tocochromanols were identified by comparison with retention times of standards purchased from Merck (>95% of purity).

### 2.5. Total Polar Compounds (TPC) Analysis

Total polar compounds in the oil were analyzed according to the AOCS Official Method 982.27 [41]. The oil sample was dissolved in toluene and applied to a glass column packed with silica gel with 5% of water (silica gel 60, 63–200 μm, Sigma-Aldrich, Poznań, Poland). A nonpolar fraction was eluted with a mixture of hexane and diisopropyl ether (82:18, *v:v*), and was collected. After evaporation of the solvent, the nonpolar fraction was weighted, and from the weight difference between the oil sample and nonpolar fraction, the polar fraction was calculated. The results were expressed as % of the content of oil.

### 2.6. Polymerized Triacylglycerols (PTG) Analysis

The polymers composition of triacylglycerol (TAG) in oil samples was determined according to AOCS Official Method 993.25 [42]. The polymer composition was analyzed using an Infinity 1290 HPLC (Agilent Technologies, Santa Clara, CA, USA) equipped with ELSD (Evaporative Light Scattering Detector) and two connected Phenogel columns (100 Å and 500 Å, 300 × 7.8 mm) (Phenomenex, Torrance, CA, USA). The conditions during the analysis were as follows: column temperature 30 °C, detector temperature 30 °C, detector pressure 2.5 bars, and injection sample volume 1 mL. The liquid phase was dichloromethane (DCM) with flow rate 1 mL/min.

### 2.7. Calculated Iodine Value (CIV)

The determination of the iodine value was conducted according to the AOCS Official Method Cd 1c-85 [43] and calculated (CIV) from fatty acid composition. The method of calculation is based on the percentage of hexadecenoic acid, octadecenoic acid, octadecadienoic acid, octadecatrienoic acid, eicosanoid acid, and docosenoic acid.

### 2.8. Statistical Analysis

All assays were replicated four times. Mean values and standard deviations were calculated with Microsoft Office Excel 2019 (Microsoft Corporation, Redmond, WA, USA). STATISTICA PL 13.3 (Dell Software Inc., Round Rock, TX, USA) were used to calculate standard errors and significant differences between means (*p* < 0.05, analysis of variance ANOVA), Tukey’s multiple range test. R software (v4.1 with packages FactoMineR v2.4 and factoextra v1.0.7) were the software used for principal components analysis (PCA).

## 3. Results and Discussion

### 3.1. Characteristics of Oil Blends

**Figure 1:**
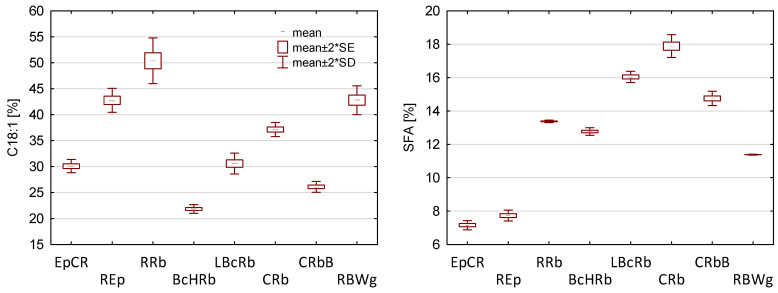

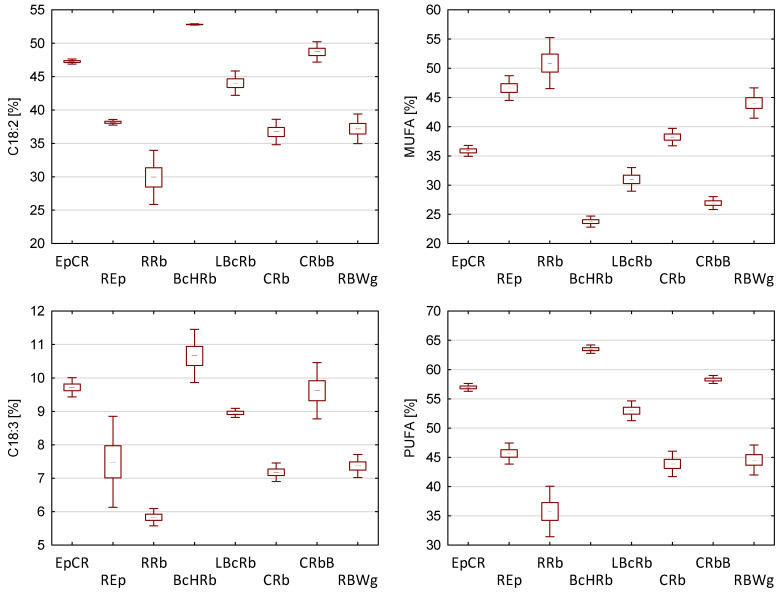
The share of main fatty acids of oil blends. SFA—saturated fatty acid, MUFA—monounsaturated fatty acid, PUFA—polyunsaturated fatty acid, SD—standard deviation, SE—standard error. Identification of oil blends is shown in Table 1.

Chromatographic analysis of the fatty acid composition confirmed that the prepared oil blends were characterized by an appropriate ratio of omega 6 to omega 3 fatty acids, and the ω6/ω3 ratio ranged from 4.86 to 5.13 (Table 1). The dominant fatty acids were oleic acid (C18:1, *n*-9) and linoleic acid (LA, C18:2, *n*-6). Among the *n*-3 fatty acids, α-linolenic acid (ALA) constituted the highest percentage in the fatty acid profile. The contents of C18:1, 18:2, and 18:3 fatty acids, as well as SFA, MUFA, and PUFA in oil blends are presented in Figure 1. Long-chain *n*-3 polyunsaturated fatty acids, especially eicosapentaenoic acid (EPA, 20:5) and docosahexaenoic acid (DHA, 22:6), have several health benefits reported in the literature, especially in relation to cardiovascular and inflammatory diseases [13,16]. There is evidence of a reduction in cardiovascular disease when consuming high doses of EPA [44], as well as a significant effect of DHA on mental health and cognitive functions [45]. The main source of them in the human diet are fish and supplements, e.g., fish oil. In the case of plant-based diets, the supply of DHA and EPA is negligible, which may cause negative health effects [12,17,18]. However, it is possible to synthesize them de novo from a plant-derived precursor (ALA) in the *n*-3 fatty acid metabolic pathway [46,47]. A sufficient high intake of ALA in the diet can be a viable substitute for marine sources and protect against deficiencies [48].

The degenerative changes in unsaturated fatty acids can be prevented by antioxidants naturally occurring in oils, in particular tocochromanols, which include tocopherols, tocotrienols, and plastochromanol-8 (PC-8) [49]. The total content of tocochromanols in the analyzed oil blends was in the following order: RBWg > EpCR > CRb > REp > BcHRb > RRb > CRbB > LBcRb (Table 2). The highest content of tocopherols was recorded for RBWg, amounting to 90.5 mg/100 g of oil, while the lowest content, amounting to 14.34 mg/100 g, for LBcRb. In the case of tocotrienols, the highest content was recorded for CRb (24.12 mg/100 g), while in EpCR and REp no presence of these compounds was detected. In vitro studies of antioxidant activity indicate that δ-tocopherol is the most active and α-tocopherol has the lowest activity. It is worth noting that in in vivo studies, the activity of tocopherols is opposite [50]. PC-8 is characterized by much higher antioxidant activity [51], as much as 1.5 times greater than the activity of α-tocopherol. The highest PC-8 content was found in REp and EpCR, while BcHRb and LBcRb did not contain PC-8.

The oxidative stability of oils largely depends on the degree of unsaturation of fatty acids. The degree of unsaturation can be determined by calculating iodine value (CIV), the greater CIV, the greater the unsaturation, and the greater the susceptibility to oxidation [52]. RRb was characterized by the lowest degree of unsaturation. The highest CIV was characteristic for BcHRb sample (Table 2).

### 3.2. Tocochromanols

α-, β-, γ-, δ-tocopherols and α-, β-, γ-, δ-tocotrienols are fat-soluble compounds that make up the vitamin E group [53]. Detailed characteristics of the content of tocopherols and tocotrienols, as well as changes in their content after thermal treatment at 170 and 200 °C, are presented in Table 3. The main tocopherol homologs found in unheated oils were γ- and α-tocopherol. Their content ranged from 9.45 to 40.89 mg/100 g of oils, and from 1.7 to 51.19 mg/100 g of oils, respectively. They constituted 75 to 98.9% of all tocochromanols in the EpCR, REp, RRb, BcHRb, and RBWg oils. In the three remaining samples (LBcRb, CRb, RbB), tocopherols constituted only 40 to 50%. The remaining 50–60% consisted primarily of β- and γ-tocotrienols. Their content ranged from 3.04 to 15.59 mg/100 g of oil and from 2.13 to 19.98 mg/100 g of oil, respectively. The high content of tocotrienols results from the presence of wheat germ oil and black cumin oil in these blends, which are characterized by a high content of tocotrienols [54,55].

Rapid losses of all tocopherols and tocotrienols were observed during heating, both at 170 °C and 200 °C. Only in the case of RBWg, the high content of all tocopherols was observed in unheated and heated samples. Excluding the RBWg oil sample, the highest loss of tocochromanols was characteristic of α-tocopherol and α -tocotrienol. Heating the samples at 170 °C resulted in a complete loss of these homologs. The only exceptions were the EpCR and CRbB samples. However, the content of these compounds in heated samples was only from 1 to 6% of the original content in unheated oils. A similar phenomenon was observed for β-tocopherols. However, its original content was very low and ranged from 0.04 to 0.58 mg/100 g of unheated oil. For other tocochromanols (γ-and δ-tocopherol as well as β- and γ-tocotrienols) their presence was also found in samples heated both at 170 and 200 °C. However, the content of homologs of individual tocochromanols was drastically reduced. Barrera-Arellano et al. [56] also described the rapid degradation of tocopherols as a result of high temperature. In their research, during heating at 180 °C for 2, 4, 6, 8, and 10 h, they analyzed changes in the content of tocopherols, indicating that the higher the loss of these compounds, the greater the content of polymeric triacylglycerols in the heated oil. As in the case of tocopherols, the content of tocotrienols also decreased significantly after the heating process.

The tocochromanols content is also important from a nutritional point of view. Each of their isoforms is biologically active, and the highest bioavailability and activity in the body is characterized by α-tocopherol. In addition to the antioxidant activity of all vitamin E isoforms, anti-proliferative, pro-apoptotic, anti-angiogenic, and anti-inflammatory effects are also described [57]. On the one hand, the high content of tocochromanols can protect the oils against degenerative changes, and on the other hand, they are nutrients important for health.

### 3.3. Formation of Polar Compounds during Heating

Heat treatment of oil, e.g., during frying food products, causes the formation of many polar compounds. Their type and number depend not only on the properties of a given oil, but also on the fried product, its weight and size, time and temperature of frying, and the presence of antioxidants. The content of total polar compounds in oils is a good indicator of degenerative fat changes [35,58]. The highest content of TPC in oils before the heating process was demonstrated for RRb (5.85%), and the lowest for CRb (1.98%) (Table 4). All samples showed a significant increase of TPC during heating, and the increase was more intense the higher the temperature was used. However, in none of the heated samples, the content of TPC exceeded the limit, which in many countries is defined at 24–25%. During the heating of the oil samples at 170 °C, the content of TPC ranged from 4.75 to 9.85%, in samples CRb and REp, respectively. In oil samples heated at 200 °C, the TPC content ranged from 8.45% (CRb) to 15.74% (REp). In most of the oil blends subjected to heating, the TPC content was related to the original TPC content of the unheated oil. The higher initial TPC content led to higher levels of this index after the heating process. The average increase in the content of the polar fraction in the heated samples was 1.9 times and 3.1 times, for the samples heated at 170 °C and 200 °C, respectively. The greatest increase in TPC level was observed in REp and CRb samples heated at 170 °C The TPC content was 2.3 times higher than that of the unheated sample. During heating at 200 °C, the highest increase was characteristic for the mixture of Camelina oil and Rice bran oil (CRb) and was 4.3 times. However, we must remember that despite the highest increase in TPC, the CRb sample was characterized by the lowest content of polar compounds, both in the unheated mixture and after heating at any temperature. The level of TPC in unheated oils may depend both on the raw material used and the method and conditions of oil pressing. The increase in TPC content during heating is the result of the transformation of fatty acids in the process of oxidation, hydrolysis, and thermal polymerization, which we can observe during frying process or heating oils [32,59]. However, the final content of polar compounds depends not only on the fatty acid profile of the oil but also on the content of antioxidants (tocochromanols, polyphenols) and other protective substances such as plant sterols. The stability of oils depends not only on the presence of these compounds in the oil but also on their concentration, structure, and synergistic interaction [60,61]. The results shown in Table 4 may confirm this thesis. The samples with the highest content of tocotrienols and average tocopherol content (LBcRb, CRb, and CRbB) had a lower content of TPC after the end of heating. When the sample contained a very high content of tocopherols (4 times higher) and a small amount of tocotrienols (RBWg sample), a similar result was obtained. When the oil samples contained only the average content of tocopherols and no tocotrienols (EpCR and Rep), the samples were characterized by the highest level of TPC. According to the data published by Seppanen et al. [60], tocotrienols are characterized by a higher antioxidant activity than tocopherols, and their different isoforms also show different activity, e.g., γ-tocotrienol has a higher antioxidant activity than α-tocotrienol [62]. The high content of tocopherol in the analyzed oil blends was as effective in protecting the oils against degenerative changes as the smaller amounts of tocotrienols. Moreover, synergistic interactions between tocochromanols are also possible, enhancing the protective properties of these compounds [63]. Nogala-Kałucka et al. [64] indicate that the antioxidant activity of tocochromanols may be increased during synergistic interactions with phenolic compounds that are also present in cold-pressed oils [7].

### 3.4. Polymerized Triacylglycerols (PTG)

Polymerization of triacylglycerols is one of the undesirable reactions that occur in oils during thermal treatment. As a result of this reaction, compounds with a high molecular weight (ranging from 692 to 1600 Daltons) are formed by a combination of -C-C-, -C-O-C-, and -C-O-O-C- bonds [65], which remain in the oil and are absorbed into the fried food, becoming part of the human diet. Dimerization and polymerization during the frying process are radical reactions. Radicals that are formed in these processes have a negative effect on oxidative stress in the intestine and their quantity may be associated with many metabolic diseases [66]. In addition, the polymers formed during frying are oxygen-rich, and oxidized polymer compounds accelerate further oxidation of the oil [67]. The high content of polar compounds also increases the viscosity of the oils, extends the frying process, and increases the fat content of fried food [68].

**Figure 2:**
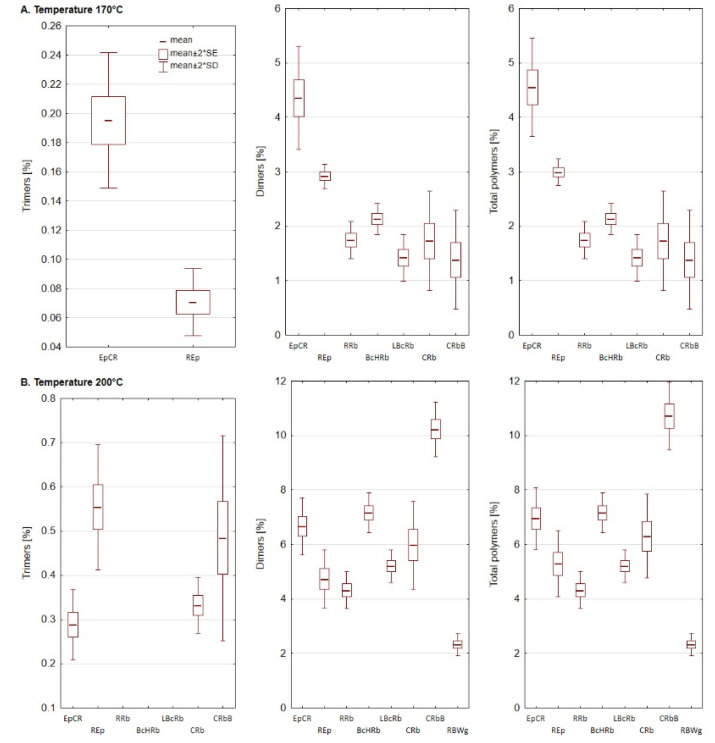
The share of dimers and trimers of triacylglycerols (TAG) in samples of oil blends heated at 170 (**A**) and 200 °C (**B**). SD—standard deviation, SE—standard error. Composition of blends is shown in Table 1.

PTG were not observed in not heated oils. The formation of polymers is significantly influenced, among others, by the temperature of the thermal treatment, but also by the heating time and the composition of fatty acids [69]. High PUFA content in oils significantly increases the reaction rate of the PTG forming process [70]. The increase of the content of polymers was characteristic for samples heated at 170 and 200 °C, and the content of individual polymers and their total content depended on the type of sample and the heating temperature (Figure 2). While the blends were heated to 170 °C, dimers were observed in 7 out of 8 oil samples. No dimers were found in the mixture of rapeseed, black cumin, and wheat germ oils (RBWg). In the remaining oils, dimers constituted from 1.38% to 4.36%. The lowest share of TAG dimers was characteristic for the LBcRb oil sample and the highest for EpCR. When the oil blends were heated at 200 °C, TAG dimers were present in all samples. Increasing the temperature of process also increased the content of this polymer fraction in oils. The share of TAG dimers in these samples ranged from 2.33% (RBWg) to 10.23% (CRbB). Comparing the results to samples heated at 170 °C the increase was 1.53 to 7.4 times. The lowest increase was observed in the EpCR and REp samples, and it was 1.53 and 1.62 times, respectively. The highest increase was characteristic of the CRbB sample (10.23 times). TAG trimers were only observed in a small number of samples. In the samples heated at 170 °C, trimers were found only in two oil blends, EpCR and REp. At a temperature of 200 °C, trimers were observed in four samples, EpCR, REp, CRb, and CRbB. The higher temperature also resulted in a higher proportion of trimers in total polymers. The highest trimer level was observed in the REp oil sample heated at 200 °C.

The lowest content of PTG in the blend of oil containing rapeseed, black cumin, and wheat germ oils (RBWg) may result from the highest content of tocochromanols in not heated oil sample. Due to the very high content of tocopherols (90.5 mg/100 g of oil) and PC-8 (1.03 mg/100 g of oil), this sample contained 1.7 to 2.6 times more tocochromanols than the other samples. The content of tocochromanols in RBWg samples was 94.24 mg/100 g of oil. In their research, Lampi and Kamal-Eldin [71] analyzed the influence of α- and γ-tocopherols on the inhibition of the polymerization process. They showed that γ-tocopherol is characterized by much higher effectiveness as an antipolymerization inhibitor. It is also argued that γ-tocotrienol has a higher antioxidant activity than α-tocotrienol, and that tocotrienols are better antioxidants than the corresponding tocopherols [62]. Therefore, the process of PTG formation is influenced not only by the PUFA contained in the oil blends but also by antioxidants that inhibit the polymerization process.

### 3.5. Principal Components Analysis

**Figure 3:**
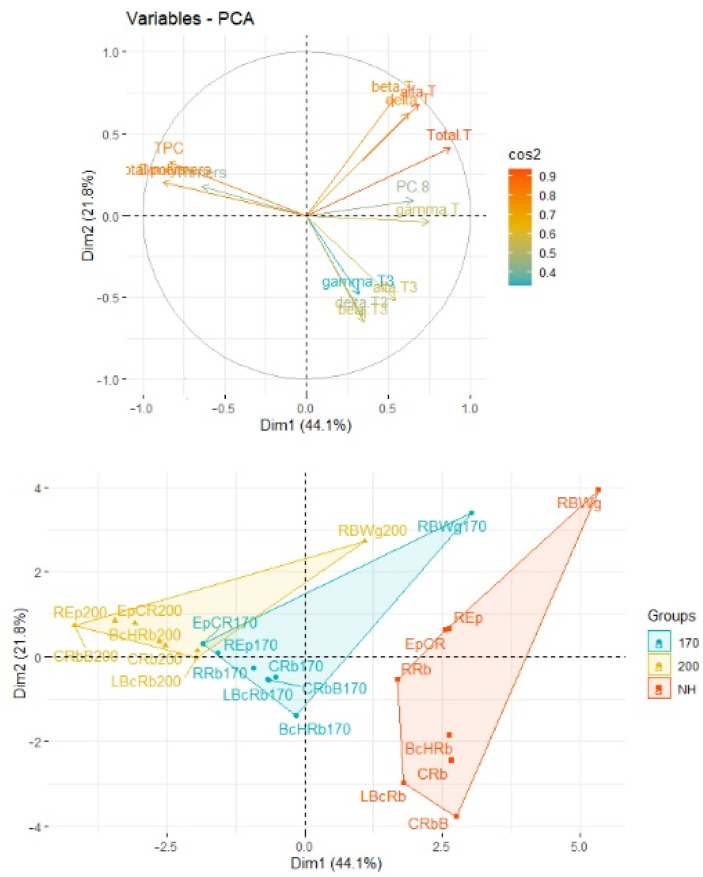
Principal component analysis (PCA) of the loadings plot and the score plot of data from α-, β-, γ-, and δ-tocopherols and tocotrienols, plastochromanol-8 (PC-8), total polar compounds (TPC), and dimers, and trimers of triacylglycerol (TAG) in oil blends heated at 170 °C and 200 °C. NH- not heated, 170 and 200—heated samples.

The principal component analysis (PCA) was applied to observe possible clusters in the oil blends heated at 170 °C and 200 °C. The first two principal factors accounted for 65.9% (Dim1 = 44.1% and Dim2 = 21.8%) of the total variation. The PCA results showed differences between the individual oil non heated samples and heated at 170 °C and 200 °C (Figure 3). Factor 1 was mainly correlated with the total tocopherols content (r = 0.877) and γ-tocopherol content (r = 0.744). It was also negatively correlated with the content of the total polymer (r = −0.879), dimers (r = −0.878), and total polar compounds content (r = −0.841). Factor 2 was mainly correlated with the β-tocopherol content (r = 0.709). The data shown in the score plot are divided into three groups. The first group (red) includes samples of unheated oil blends. The two remaining groups contain samples heated at 170 °C (blue) and 200 °C (yellow). For unheated samples, two subgroups and one outlier were observed. The first subgroup contained 3 samples (REp, EpCR, and RRb) and was located close to the x axis. These samples differed from the others with a high content of α-tocopherol (16.61–20.62 mg/100 g of oil) and total tocopherols content (43.84−61.25 mg/100 g of oil). In the second subgroup, which was located under the x axis and to the right of the y axis, there were 4 samples (BcHRb, LBcRb, CRb, and CRbB). The content of α-tocopherol ranged from 1.70 to 9.71 mg/100 g of oil. The outlier sample, which was located high above the x axis and to the right of the y axis, was a blend of oil mixed with wheat germ oil. This blend was characterized by the highest content of α-tocopherol (51.19 mg/100 g of oil) and the total content of tocopherols (106.50 mg/100 g of oil). In the two remaining groups (heated oils), much smaller distances between individual samples were observed. Oil samples heated at 170 °C are located close to the center of the plot on the x axis. Samples heated at 200 °C are located above the x axis and to the left of the y axis. Within the group, the oil samples were most differentiated by the content of TAG dimers and the content of γ-tocopherol. As before, in each group, outliers were observed, which were oil blends with wheat germ oil. In these samples, the degradation of tocopherols and the increase of TAG polymers and the polar fraction content were slower. However, the trend of the outliers was comparable to that of other oils. On the score plot, the movement of the samples from the left to the right side of the graph was observed.

## 4. Conclusions

Enriching the human diet with vegetable oils is one of the ways to counteract the consumption of excessive amounts of animal fats with high concentrations of saturated fatty acids. Vegetable oils provide many essential substances such as unsaturated fatty acids, especially PUFA, but also fat-soluble vitamins, antioxidants, and plant sterols. The ratio of omega-6 to omega-3 fatty acids is also very important from a health point of view. However, the presence of a high PUFA content makes oils susceptible to oxidation, especially when there are exposed to high temperatures.

Heating oils with a programmed ratio of ω6/ω3 fatty acids led to their thermal degradation. The degree of degradation was higher, with the higher temperature of the process being used. In addition, the degradation rate was related to the fatty acid profile and the tocochromanols content in individual blends. During the heating process, a sharp decrease in tocochromanols (tocopherols and tocotrienols) and an increase in TPC content were observed. The high temperature of heating also increased the level of polymerization products of triacylglycerols, in particular TAG dimers. The changes were related to the content of tocopherols and tocotrienols. The high level of tocopherols limited the transformations in a similar way as the much lower share of both tocopherols and tocotrienols in the oil. This may suggest the possibility of obtaining appropriate oils also with a lower content of antioxidants, however, coming from different groups and using the synergistic phenomenon. Regardless of the observed changes, none of the prepared oil blends exceeded the limit value of the TPC content, maintaining the programmed ratio of ω6 to ω3 acids. This confirms the possibility of creating and using oils with a nutritious ratio of ω6/ω3 fatty acids in nutrition and food production technology.

The use of developed oil blends can be used both to form plant-based meat analogs with high nutritional value and to improve the quality of conventional products. Further research on oil blends may deepen our knowledge about the mechanisms of MUFA and PUFA degradation during technological processes and develop effective methods of their prevention.

## Figures and Tables

**Table 1:** Composition of oil blends.

No.	Code	Type of Oil			Share [%]			Ratio ω6/ω3
1	EpCR	Evening primrose oil	Camelina oil	Rapeseed oil	50	10	40	4.86:1
2	REp	Rapeseed oil	Evening primrose oil		65	35	-	5.11:1
3	RRb	Rapeseed oil	Rice bran oil		45	55	-	5.13:1
4	BcHRb	Black cumin oil	Hemp oil	Rice bran oil	25	55	20	4.96:1
5	LBcRb	Linseed oil	Black cumin oil	Rice bran oil	15	45	40	4.92:1
6	CRb	Camelina oil	Rice bran oil		12	88	-	5.11:1
7	CRbB	Camelina oil	Rice bran oil	Black cumin oil	18	10	72	5.07:1
8	RBWg	Rapeseed oil	Black cumin oil	Wheat germ oil	50	30	20	5.05:1

**Table 2:** Content of tochochromanols [mg/100 g of oil] and Calculated Iodine Value (CIV) of oil blends.

Type of Blends ^1^	PC-8	Total Tocopherols	Total Tocotrienols	CIV
EpCR	1.15 ± 0.06 ^b^	60.25 ± 0.56^a^	nd	137.69 ± 0.29 ^d^
REp	1.38 ± 0.03 ^d^	57.16 ± 0.66^a^	nd	125.51 ± 1.24 ^bc^
RRb	0.40 ± 0.01 ^a^	37.84 ± 1.09^e^	11.47 ± 0.36 ^c^	110.81 ± 1.96 ^a^
BcHRb	nd	44.92 ± 0.63 ^f^	7.17 ± 0.04 ^b^	139.67 ± 0.56 ^d^
LBcRb	nd	14.34 ± 0.18 ^b^	20.12 ± 0.17 ^d^	126.32 ± 0.54 ^c^
CRb	0.8 ± 0.01 ^c^	26.19 ± 0.27 ^d^	24.12 ± 0.07 ^f^	115.15 ± 1.37 ^a^
CRbB	0.50 ± 0.12 ^a^	18.09 ± 0.42 ^c^	21.46 ± 0.36 ^e^	132.60 ± 0.26 ^e^
RBWg	1.03 ± 0.03 ^b^	90.5 ± 2.52 ^g^	2.71 ± 0.03 ^a^	121.52 ± 1.26 ^b^

^1^ Composition of blends as shown in Table 1. nd—not detected. PC-8—plastochromanol 8. CIV—Calculated Iodine Value. Values are means of four determinations ± SD. Means in the same column, followed by different small letters, indicate significant differences (*p* < 0.05) between samples.

**Table 3:** Changes of tocochromanol contents [mg/100 g of oil] during heating oil blends.

Tocopherols												
Type of Blends ^1^	α-T			β-T			γ-T			δ-T		
	Not Heated	Heating Temperature		Not Heated	Heating Temperature		Not Heated	Heating Temperature		Not Heated	Heating Temperature	
		170 [°C]	200 [°C]		170 [°C]	200 [°C]		170 [°C]	200 [°C]		170 [°C]	200 [°C]
EpCR	18.62 ± 0.24 ^cd^	0.24 ± 0.04 ^a^	nd	0.06 ± 0.01 ^a^	nd	nd	40.89 ± 0.35 ^b^	4.50 ± 0.04 ^d^	3.88 ± 0.04 ^e^	0.69 ± 0.02 ^b^	0.42 ± 0.06 ^c^	0.39 ± 0.01 ^c^
REp	20.61 ± 0.36 ^d^	nd	nd	0.04 ± 0.00 ^a^	nd	nd	35.86 ± 0.31 ^f^	0.22 ± 0.01 ^b^	nd	0.64 ± 0.01 ^ab^	0.24 ± 0.01 ^b^	0.16 ± 0.01 ^b^
RRb	16.61 ± 0.45 ^c^	nd	nd	0.23 ± 0.01 ^a^	nd	nd	20.61 ± 0.66 ^e^	1.21 ± 0.08 ^a^	0.14 ± 0.02 ^ab^	0.40 ± 0.03 ^ab^	0.05 ± 0.00 ^a^	nd
BcHRb	3.67 ± 0.18 ^ab^	nd	nd	0.19 ± 0.01 ^a^	nd	nd	39.64 ± 0.28 ^b^	8.41 ± 0.05 ^e^	2.08 ± 0.05 ^d^	1.42 ± 0.15 ^c^	0.40 ± 0.02 ^c^	nd
LBcRb	4.33 ± 0.01 ^b^	nd	nd	0.40 ± 0.07 ^a^	nd	nd	9.47 ± 0.21 ^c^	0.81 ± 0.04 ^ab^	0.57 ± 0.02 ^c^	0.14 ± 0.03 ^a^	nd	nd
CRb	9.71 ± 0.01 ^e^	nd	nd	0.30 ± 0.00 ^a^	nd	nd	15.87 ± 0.23 ^a^	2.83 ± 0.11 ^c^	2.28 ± 0.04 ^d^	0.31 ± 0.06 ^ab^	0.14 ± 0.02 ^ab^	0.11 ± 0.1 ^a^
CRbB	1.70 ± 0.03 ^a^	0.07 ± 0.00 ^a^	nd	0.58 ± 0.06 ^a^	nd	nd	15.57 ± 0.35 ^a^	1.18 ± 0.01 ^a^	0.41 ± 0.01 ^bc^	0.24 ± 0.01 ^ab^	0.11 ± 0.04 ^ab^	nd
RBWg	51.19 ± 1.44 ^f^	48.58 ± 0.04 ^b^	42.66 ± 0.09	13.75 ± 0.38 ^b^	11.71 ± 0.04	10.38 ± 0.18	18.60 ± 0.35 ^d^	14.39 ± 0.45 ^f^	9.45 ± 0.28 ^f^	6.96 ± 0.35 ^d^	4.14 ± 0.06 ^d^	1.12 ± 0.01 ^d^
**Tocotrienols**												
**Type of Blends ^1^**	**α-T3**			**β-T3**			**γ-T3**			**δ-T3**		
	**Not Heated**	**Heating Temperature**		**Not Heated**	**Heating Temperature**		**Not Heated**	**Heating Temperature**		**Not Teated**	**Heating Temperature**	
		**170 [°C]**	**200 [°C]**		**170 [°C]**	**200 [°C]**		**170 [°C]**	**200 [°C]**		**170 [°C]**	**200 [°C]**
EpCR	nd	nd	nd	nd	nd	nd	nd	nd	nd	nd	nd	nd
REp	nd	nd	nd	nd	nd	nd	nd	nd	nd	nd	nd	nd
RRb	0.50 ± 0.02 ^a^	nd	nd	1.83 ± 0.04 ^c^	nd	nd	9.14 ± 0.38 ^c^	0.515 ± 0.02 ^a^	nd	nd	nd	nd
BcHRb	1.18 ± 0.03 ^c^	nd	nd	3.40 ± 0.08 ^a^	1.12 ± 0.08 ^b^	0.54 ± 0.04 ^b^	2.09 ± 0.00 ^a^	0.45 ± 0.03 ^a^	0.09 ± 0.01 ^a^	0.51 ± 0.09 ^c^	0.53 ± 0.03	nd
LBcRb	2.11 ± 0.11 ^e^	nd	nd	11.14 ± 0.04 ^d^	1.62 ± 0.08 ^c^	1.56 ± 0.08 ^d^	6.70 ± 0.22 ^b^	0.58 ± 0.06 ^a^	0.43 ± 0.01 ^b^	0.18 ± 0.03 ^a^	nd	nd
CRb	0.83 ± 0.03 ^b^	nd	nd	3.05 ± 0.02 ^a^	nd	nd	19.98 ± 0.12 ^d^	2.26 ± 0.16 ^c^	2.15 ± 0.11 ^c^	0.27 ± 0.00 ^ab^	nd	nd
CRbB	3.41 ± 0.01 ^f^	0.22 ± 0.01	nd	15.59 ± 0.33 ^e^	2.12 ± 0.08 ^d^	1.33 ± 0.01 ^c^	2.13 ± 0.01 ^a^	1.05 ± 0.00 ^b^	nd	0.33 ± 0.01 ^b^	nd	nd
RBWg	1.44 ± 0.01 ^d^	nd	nd	1.28 ± 0.04 ^b^	0.65 ± 0.00 ^a^	0.34 ± 0.03 ^a^	nd	nd	nd	nd	nd	nd

^1^ Composition of blends as shown in Table 1. nd—not detected. Values are means of four determinations ± SD. Means in the same row, followed by different small letters indicate significant differences (*p* < 0.05) between samples in the same heating temperature.

**Table 4:** Changes of total polar compounds (TPC) during heating oil blends [%].

Type of Blends ^1^	Not Heated	Heating Temperature	
		170 [°C]	200 [°C]
**EpCR**	4.75 ± 0.07 ^aB^	8.85 ± 0.07 ^bD^	13.54 ± 0.27 ^cE^
REp	4.21 ± 0.17 ^aB^	9.85 ± 0.17 ^bE^	15.74 ± 0.04 ^cF^
RRb	5.85 ± 0.16 ^aD^	7.52 ± 0.25 ^bA^	10.85 ± 0.07 ^cB^
BcHRb	3.47 ± 0.00 ^aA^	6.95 ± 0.38 ^bA^	11.74 ± 0.17 ^cC^
LBcRb	3.25 ± 0.13 ^aA^	6.04 ± 0.06 ^bB^	9.74 ± 0.38 ^cA^
CRb	1.98 ± 0.14 ^aC^	4.57 ± 0.07 ^bC^	8.45 ± 0.10 ^cD^
CRbB	4.31 ± 0.33 ^aB^	7.05 ± 0.06 ^bA^	11.21 ± 0.11 ^cBC^
RBWg	3.41 ± 0.01 ^aA^	5.85 ± 0.14 ^bB^	9.85 ± 0.20 ^cA^

^1^ Composition of blends as shown in Table 1. Values are means of four determinations ± SD. Means in the same row, followed by different small letters indicate significant differences (*p* < 0.05) between the same samples with different heating temperatures. Means in the same column, followed by different capital letters, indicate significant differences (*p* < 0.05) between samples in the same heating temperature.

## Data Availability

All data generated or analyzed during this study are included in this published article.

## Abbreviations

ALA—α-linolenic acid, 18:3, *n*−3; DHA—docosahexaenoic acid, 22:6, *n*−3; EPA—eicosapentaenoic acid, 20:5, *n*−3; LA—linoleic acid, C18:2, *n*-6; -8; SFA—saturated fatty acids; MUFA—monounsaturated fatty acids; PUFA—polyunsaturated fatty acids; CIV—calculated iodine value; TPC—total polar compounds; TAG—triacylglycerol; PTG—polymerized triacylglycerols; α-T—α-Tocopherol; α-T3—α-Tocotrienol; β-T—β-Tocopherol; β-T3—β-Tocotrienol; γ-T—γ-Tocopherol; γ-T3—γ-Tocotrienol; δ-T—δ-Tocopherol; δ-T3—δ-Tocotrienol; PC-8—plastochromanol.
